# Computing away the magic?

**DOI:** 10.7554/eLife.01135

**Published:** 2013-08-06

**Authors:** Michael Levine

**Affiliations:** 1 **Michael Levine** is at the Center for Integrative Genomics, Division of Genetics, Genomics, and Development, Department of Molecular and Cell Biology, University of California, Berkeley, Berkeley, United Statesmlevine@berkeley.edu

**Keywords:** transcriptional regulation, logistic regression, fly embryo, developmental patterning, positional information, even skipped, *D. melanogaster*

## Abstract

Computer simulations and quantitative imaging of *Drosophila* embryos have been used to recreate the dynamic activities of a complex transcriptional enhancer.

**Related research article** Ilsley GR, Fisher J, Apweiler R, DePace AH, Luscombe NM. 2013. Cellular resolution models for *even skipped* regulation in the entire *Drosophila* embryo. *eLife* **2**:e00522. doi: 10.7554/eLife.00522

**Image** Quantification of transcription factors can be used to train computational models of gene expression in *Drosophila* embryos

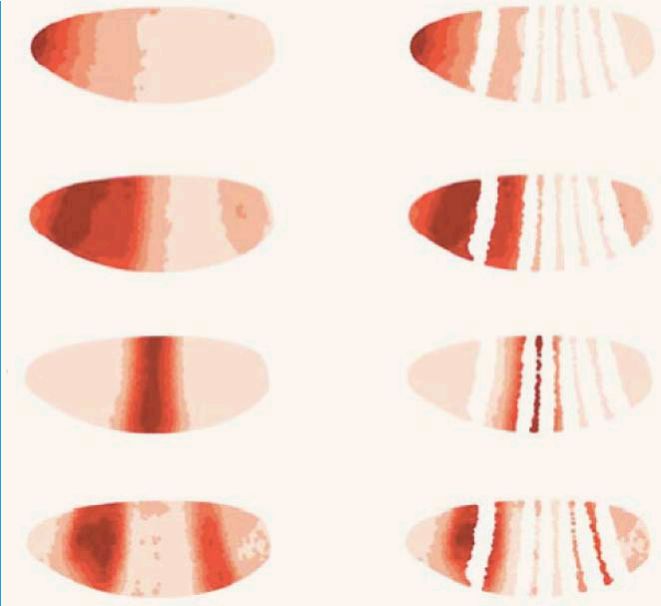

Multicellular organisms employ a variety of mechanisms to ensure that genes are expressed at the right time and place throughout their life cycles. The transcription of DNA into RNA is augmented by activators and diminished by repressors. Both classes of regulatory proteins bind to specific sequences contained within enhancers, which are the key agents of gene regulation in higher organisms. Elucidating how enhancers work is critical for understanding gene regulation in development and disease.

It is over 30 years since Banerji and Schaffner discovered that enhancers can be physically separate from the genes they regulate (Banerji et al., 1981). Enhancers can map quite far—1 million base pairs or more—from their target genes (Amano et al., 2009). This action at a distance is a defining property of complex organisms, and contrasts with what happens in simple bacteria, where most activator and repressor binding sites are found quite close to their target genes (see, e.g., Levine and Tjian, 2003).

One of the most widely studied enhancers is the *eve* stripe 2 enhancer in the fruit fly *Drosophila melanogaster* (Small et al., 1992). The body of the *Drosophila* embryo is made up of 14 segments, and a gene called *eve* (*even-skipped*) is expressed in the even-numbered segments, giving rise to a distinctive pattern of seven stripes (Figure 1A). It was initially thought that the long-range diffusion of morphogens (Turing, 1952)—signaling molecules that influence tissue development through their formation of concentration gradients—coordinated the expression of all seven *eve* stripes (Meinhardt, 1986). The discovery that *eve* stripe 2 had its own dedicated enhancer led one researcher to complain of the ‘inelegance’ of such a mechanism (Akam, 1989). However, we have now come full circle: I cannot help but complain that the new models for the regulation of *eve* expression described by Nicholas Luscombe and co-workers in *eLife* seem to strip the mystique from the *eve* stripe 2 enhancer (Ilsley et al., 2013).

**Figure 1.**
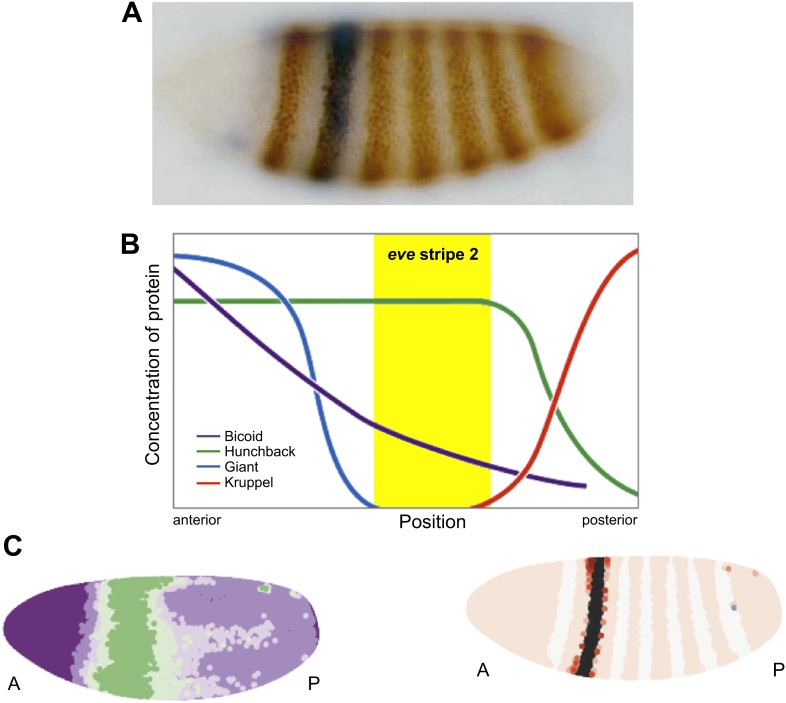
Regulation of *eve* stripe 2. The gene *eve* is expressed in the even-numbered body segments of *Drosophila* embryos, giving rise to a distinctive pattern of stripes. **A**, Transgenic embryo expressing an eve.2>lacZ fusion gene. The endogenous *eve* stripes are stained brown, while stripe 2 is stained blue (Small et al., 1992). **B**, The transcription factors Krüppel and Giant (repressors) and Bicoid and Hunchback (activators) are expressed in distinct patterns along the *Drosophila* embryo, and their combined effects dictate the position of *eve* stripe 2 (Watson et al., 2014). **C**, Computer simulations can be used to model the expression gradient of Bicoid (left) and the resulting effect on the position of *eve* stripe 2 (right). A, anterior; P, posterior.

The stripe 2 enhancer is regulated by four different transcription factors in the early *Drosophila* embryo—two activators, Bicoid and Hunchback; and two repressors, Giant and Krüppel (Small et al., 1992). There are 12 binding sites for these transcription factors distributed over the length of the enhancer, and the combined effects of these four proteins dictate the location of the second *eve* stripe (Figure 1B). In principle, Bicoid and Hunchback can activate the *eve* stripe 2 enhancer in the entire anterior half of the embryo (from the head to the anterior thorax); however, localized repressors—Giant and Krüppel—delineate *eve* expression within the stripe 2 domain.

Luscombe and co-workers—including Garth Ilsley as first author—investigated how these four transcription factors produce the stripe 2 expression pattern (Figure 1B), by combining quantitative imaging with computer simulations of different mathematical models. They used this same approach to model the enhancer that regulates stripes 3 and 7, but for simplicity I will restrict my discussion to stripe 2. The resulting models provide new insights into the mechanisms of stripe formation during development. First, IIsley et al. argue that the order of the Bicoid, Hunchback, Giant and Krüppel binding sites is unlikely to be important for stripe 2 expression. They base this on the observation that models in which the effects of activators can simply be added to those of repressors are sufficient to produce the stripe 2 pattern, and there is no need to assume that activators bound to adjacent sites cooperate with each other to augment their activities. Moreover, there is no indication of nonlinear effects such as ‘repression dominance’, whereby repressors downregulate transcription more than activators upregulate it (Arnosti et al., 1996). Rather, the models call for a simple balance between the effects of activators and those of repressors.

The most interesting implication of this work is that Bicoid might not function solely as an activator (Driever et al., 1989; Struhl et al., 1989). Luscombe and co-workers were able to achieve more faithful simulations of the stripe 2 expression pattern by assuming that Bicoid, which is most abundant in the anterior region of the embryo and gradually declines in concentration towards the posterior end, acts as both an activator and a repressor. Ilsley et al. propose that high levels of Bicoid repress expression of stripe 2 in anterior regions, while lower levels in the more central regions activate its expression (Figure 1C).

The idea that a transcription factor can mediate both activation and repression is not new. However, this is the first time that such a dual mechanism has been suggested for Bicoid, the lynchpin of anterior–posterior patterning. This dual function of Bicoid can explain why *eve*, and many other segmentation genes, are silent at the anterior pole of the *Drosophila* embryo (Andrioli et al., 2002).

In summary, the *eve* stripe 2 enhancer produces an exquisite on/off pattern of expression in response to crude gradients of transcription factors, and its ability to do so has previously been explained by nonlinear interactions between proteins. By arguing against such nonlinearity, Ilsley et al. seemingly strip the magic from the stripe 2 enhancer. But is the magic really gone? How the enhancer determines whether Bicoid functions as an activator or a repressor is uncertain. Hence, I believe that the concept of the enhancer as a template for weak protein interactions is alive and well, and yes, still a mystery.
